# Development of a Prognostic Signature Based on Single-Cell RNA Sequencing Data of Immune Cells in Intrahepatic Cholangiocarcinoma

**DOI:** 10.3389/fgene.2020.615680

**Published:** 2021-02-04

**Authors:** Miao Su, Kuang-Yuan Qiao, Xiao-Li Xie, Xin-Ying Zhu, Fu-Lai Gao, Chang-Juan Li, Dong-Qiang Zhao

**Affiliations:** ^1^Department of Gastroenterology, The Second Hospital of Hebei Medical University, Shijiazhuang, China; ^2^Department of Gastroenterology, Hengshui People's Hospital, Hengshui, China; ^3^Basic Medical College, Hebei Medical University, Shijiazhuang, China; ^4^Department of Gastroenterology, The Third Hospital of Hebei Medical University, Shijiazhuang, China

**Keywords:** intrahepatic cholangiocarcinoma, single-cell RNA sequencing, immune cells, differentially expressed genes, progression

## Abstract

Analysis of single-cell RNA sequencing (scRNA-seq) data of immune cells from the tumor microenvironment (TME) may identify tumor progression biomarkers. This study was designed to investigate the prognostic value of differentially expressed genes (DEGs) in intrahepatic cholangiocarcinoma (ICC) using scRNA-seq. We downloaded the scRNA-seq data of 33,991 cell samples, including 17,090 ICC cell samples and 16,901 ICC adjacent tissue cell samples regarded as normal cells. scRNA-seq data were processed and classified into 20 clusters. The immune cell clusters were extracted and processed again in the same way, and each type of immune cells was divided into several subclusters. In total, 337 marker genes of macrophages and 427 marker genes of B cells were identified by comparing ICC subclusters with normal subclusters. Finally, 659 DEGs were obtained by merging B cell and macrophage marker genes. ICC sample clinical information and gene expression data were downloaded. A nine-prognosis-related-gene (PRG) signature was established by analyzing the correlation between DEGs and overall survival in ICC. The robustness and validity of the signature were verified. Functional enrichment analysis revealed that the nine PRGs were mainly involved in tumor immune mechanisms. In conclusion, we established a PRG signature based on scRNA-seq data from immune cells of patients with ICC. This PRG signature not only reflects the TME immune status but also provides new biomarkers for ICC prognosis.

## Introduction

Intrahepatic cholangiocarcinoma (ICC) is the second most prevalent type of primary liver cancer worldwide (Massarweh and El-Serag, 2017) and accounts for 10–15% of malignant hepatic tumors. ICC arises from bile duct epithelial cells and is characterized by insidious onset and rapid progression. The 3- and 5-year survival rates for ICC are only 30 and 18%, respectively (Bartella and Dufour, 2015). Moreover, only 30% of patients with ICC have the opportunity to undergo surgical resection, and the post-operative recurrence rate is high (Chun and Javle, 2017; Rahnemai-Azar et al., 2017). Worldwide, the morbidity and mortality of ICC have increased over the past few decades (Rizvi et al., 2018). To this end, accurately predicting prognosis is of great clinical significance. However, the existing ICC prognostic markers cannot precisely predict the prognosis because of their poor sensitivity and specificity (Qin and Song, 2020), which may greatly affect the choice of treatment strategies.

The immune system, as major components of the tumor microenvironment (TME), largely determines the development and progression of cancer by regulating various immune cells (Angell and Galon, 2013). Genetic heterogeneity means that patients with ICC sometimes suffer significantly different clinical outcomes, despite having the same condition and treatment. Immune cells play a pivotal role in the prognosis of ICC (Loeuillard et al., 2019). Single-cell RNA sequencing (scRNA-seq), a new technique allowing high-throughput sequencing analysis of the genome, transcriptome, and epigenome at the single-cell level, can reveal the gene structure and gene expression state of single cells and uncover intercell heterogeneity (Yu et al., 2018), making up for the limitations of traditional high-throughput sequencing (Nguyen et al., 2018). scRNA-seq has been successfully applied in a variety of malignant tumors (Chung et al., 2017; Peng et al., 2019) to analyze intratumor heterogeneity, monitor circulating cancer cells, and predict tumor prognosis (Navin, 2015). However, no models based on scRNA-seq have been developed to reliably predict ICC prognosis.

This study was designed to identify new biomarkers to assess the ICC tumor immune microenvironment status and accurately predict the prognosis for patients with ICC. In this study, differentially expressed genes (DEGs) were screened by comparing immune cell scRNA-seq data in ICC tissue with that in ICC adjacent tissue. The correlation between DEGs and ICC prognosis was analyzed to develop a prognostic signature.

## Materials and Methods

### Acquisition of Cell Samples and ICC Population Cohorts

**Figure 1:**
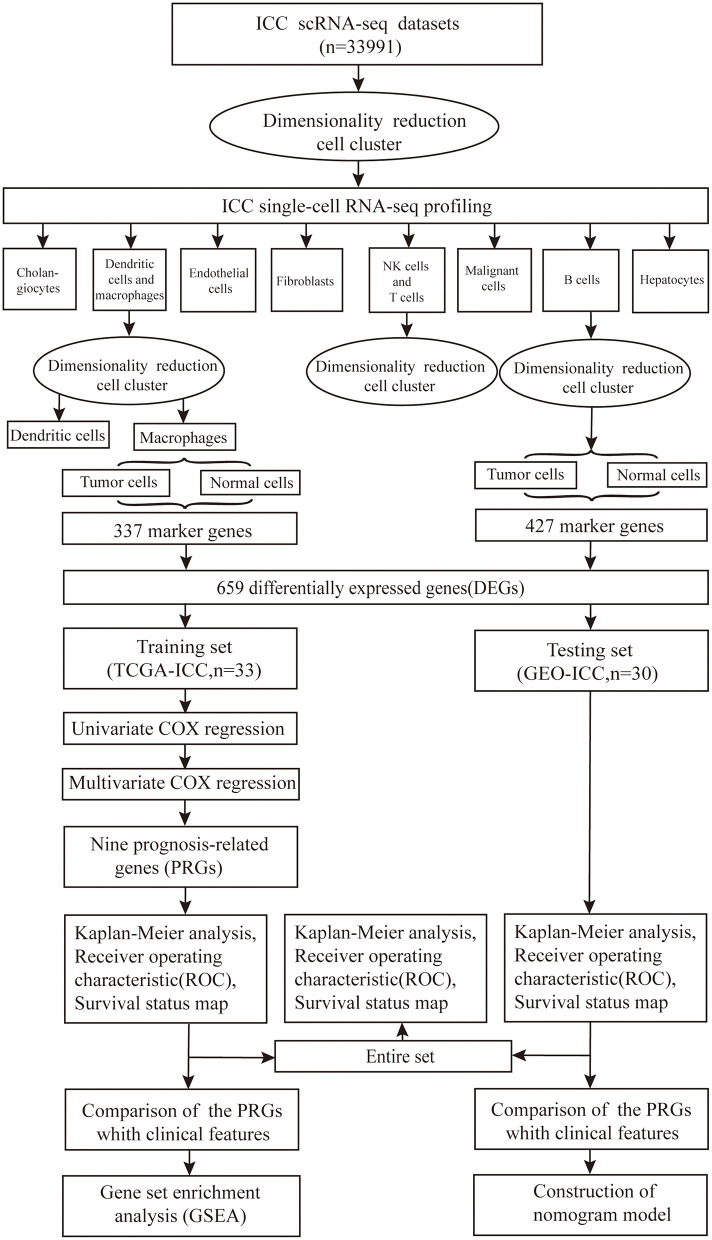
Flowchart of the study. The scRNA-seq data of 33991 cell samples were downloaded, nine prognosis-related genes (PRGs) were identified and the robustness and validity verified.

The GSE138709 single-cell transcriptome profiles of ICC and adjacent tissue cell samples were downloaded from the Gene Expression Omnibus (GEO) database. The adjacent tissue was regarded as normal tissue. ICC sample clinical information and gene expression data were downloaded from The Cancer Genome Atlas (TCGA) data portal and GEO database (GSE107943) in August 2020. All the data in this study were obtained directly from the public database, and the relevant guidelines were strictly followed, allowing ethical approval to be exempted. The workflow is shown in Figure 1.

### Processing of scRNA-Seq Data

R language scripts were written to analyze scRNA-seq data. The counts files were read into R and formatted; averages were obtained for duplicated genes, and transcriptome sequence data of ICC cells and adjacent tissue cells were merged into a matrix. We used the statistical R package “Seurat” to process the data, including data quality control, gene and cell filtration, normalization, variable gene finding, data scaling, principal component analysis (PCA), and t-distributed stochastic neighbor embedding (t-SNE) algorithms. All default parameters were left unchanged unless otherwise specified. First, the single-cell data were processed by CreateSeuratObject function (arguments: min. cells = 3, min. features = 200) to create the object. Meanwhile, cells with poor quality were excluded. Only genes detected in more than three cells and cells with more than 200 detected genes were used in the following analysis. We conducted quality control using PercentageFeatureSet function (arguments: pattern = ^∧^MT-), which could calculate gene number, gene types number, and percentage of mitochondrial genes. The correlation between sequenced genes number and sequenced genes types was calculated with FeatureScatter function. The results were also visualized. Second, to exclude non-cells or cell aggregates, subset function was used to further screen samples with the selective criteria of gene expression types of more than 500, gene expression levels of more than 1,000 and fewer than 20,000, and mitochondrial proportion restricted to <20%. The data were log-normalized with NormalizeData function, and the top 1,500 variable genes were identified using the FindVariableFeatures function (arguments: selection.method = vst, nfeatures = 1,500) for subsequent analysis. Third, we used the ScaleData function (vars.to.regress = percent.mt) to mitigate this source of variation in the dataset. PCA was performed by RunPCA function for dimension reduction. After calculation with the JackStraw function, the JackStrawPlot (dims = 1:20) and ElbowPlot functions (ndims = 40) were used to identify the number of significant principal components (PCs) to use for clustering. Through plot visualization, the top 20 PCs were selected for the next analysis. Lastly, cell populations were clustered by t-SNE algorithm. FindClusters function with resolution of 0.5 was performed, and RunTSNE function was used to generate clusters. The FindAllMarkers function (arguments: min.pct = 0.25, logfc.threshold = 0.25) was used to find markers by comparing each cluster with all others; different genes between two identities were identified using the FindMarkers function. The feature plot and heatmap visualization of gene expression were generated using the Seurat function FeaturePlot and DoHeatmap, respectively. Cell type–specific marker genes were taken from published literature (Zhang et al., 2020) and were compared with our analysis results to define the cluster type. Clusters consisting of immune cells were extracted and processed again in the same way as above, and each immune cell type was further divided into subclusters. Marker genes of each immune cell type were identified by comparing ICC subclusters with normal subclusters, and adjustment of *P*-value (adjPval) <0.05 was regarded as the cutoff criteria. The marker genes of each immune cell type were incorporated as DEGs.

### Development of a Prognostic Signature

The ICC samples from TCGA (TCGA-ICC) were regarded as the training set for prognostic signature establishment; the ICC samples from the GEO database (GEO-ICC) were used as the testing set. The testing and training sets were merged as an entire set. The testing set and the entire set were employed to validate the predictive ability of established signature. Univariate Cox regression was used to analyze the relationship between DEGs and overall survival (OS) in the training set. Finally, prognosis-related genes (PRGs) were further identified from the DEGs most associated with prognosis by multivariate Cox regression analysis. The prognostic genes risk scores were determined based on gene expression multiplied by a linear regression coefficient combination. The formula for risk score calculation for all patients was as follows:

$$\begin{array}{l} Survival\;risk\;score\left(SRS\right)=\overset{k}{\underset{i=1}{\sum }}\left(C_{i}\;*\;V_{i}\right) \end{array}$$

In the formula, *k* represents the number of mRNA, *C*_*i*_ represents the coefficient of mRNA in multivariate Cox regression analysis, and *V*_*i*_ represents the mRNA expression level.

### PRGs Signature Validation

To verify the power of the PRG signature, patients with ICC were divided into high- and low-risk groups based on the median risk scores in the training, testing, and entire sets. OS was compared in high- and low-risk groups using Kaplan–Meier analysis. Survival analysis was also conducted using each of the PRGs in the training and testing sets. Receiver operating characteristic (ROC) curve analysis based on the risk scores of PRGs was performed in the three sets with R package “survivalROC” (arguments: method = KM), and the value of the area under the curve (AUC) was determined to verify the model sensitivity and accuracy. Finally, the survival status map showed the distribution of death endpoint events based on the risk scores of PRGs.

### Comparison Between the PRG Signature and Clinical Features in the TCGA-ICC and GEO-ICC Cohorts

We used survivalROC function (arguments: method = KM) to assess the prognostic ability of the PRG signature and the clinical variables provided in the clinical data. The ability of the prognostic predictors was compared by ROC analysis, and the value of the AUC was determined for each parameter. Utilizing the generalized linear model regression algorithm, the PRG nomogram model was established through the risk score of the GEO-ICC.

### Functional Pathway Enrichment Analysis

The TCGA-ICC cohort was divided into two groups with high and low PRG risk score levels, and gene set enrichment analysis (GSEA) was performed using the PRG risk score as the phenotype.

### Statistical Analysis

Single-cell sequencing data were analyzed using the Seurat package. The ggplot2 package was used to produce the single-cell analysis graph. Cox regression analysis was performed using the glmnet and survival packages. The nomogram model was established by the rms package. The survival curve was generated by the survival package. *P* < 0.05 was regarded as statistically significant. All the statistical analyses were performed by R language, version 3.6.1.

## Results

### Profiling of scRNA-Seq and Screening of Marker Genes

**Figure 2:**
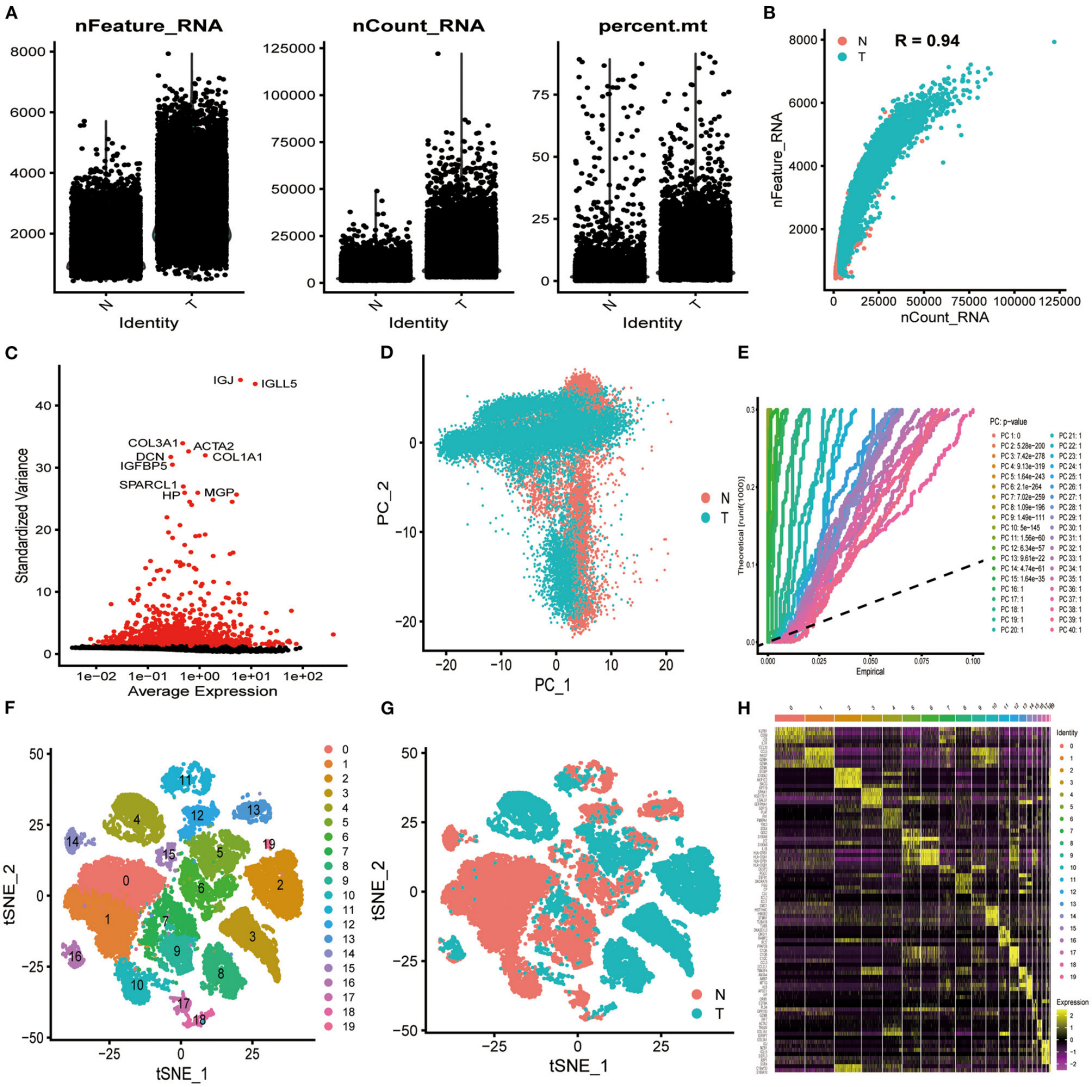
Characterization of scRNA-seq from 33,991 cells. **(A)** Quality control of scRNA-seq in ICC cell and normal cell samples. **(B)** The detected gene numbers positively correlated with sequencing depth. T represents cells derived from ICC tissue; N represents cells derived from normal cell samples. **(C)** In total, 1,500 gene symbols with significant differences across cells were identified, and the characteristic variance diagram was drawn. **(D,E)** PCA was conducted to identify the significantly available dimensions of data sets with estimated *P* value. Cells were classified by PCA. **(F)** Based on the available significant components from PCA, t-SNE algorithm was performed, and cells were further divided into 20 clusters. **(G)** Cells derived from ICC or normal tissue are shown. **(H)** The top 85 marker genes across the 20 clusters are exhibited.

In total, 33,991 cell samples that comprised 17,090 tumor cells and 16,901 normal cells from eight patients with ICC were obtained from the GEO database (Table 1). The quality control chart shows the detected gene number range, sequence count for each cell, and the percent of mitochondrial genes (Figure 2A). There was a positive association between detected gene counts and sequencing depth with Pearson *r* = 0.94 (Figure 2B). After filtering out the poor-quality cells, gene expression data from 29,263 cells were used for further analysis. The top 10 genes, including IGJ, IGLL5, COL3A1, ACTA2, and COL1A1, with significant differences across the cell samples are shown in Figure 2C. PCA was performed to preliminarily classify the cells (Figure 2D), and a Jack Straw Plot showed the *P* value distributions for each PC (Figure 2E). The dot plot shows the top 20 genes, and the top 30 significantly correlated genes were shown via heatmap (Supplementary Figures 1A,B). The scree plot displayed how much variation each PC captured from the data (Supplementary Figure 1C). Furthermore, the t-SNE algorithm was used to further precisely cluster the cells, and the samples were successfully classified into 20 clusters (Figure 2F, Supplementary Table 1). The marker genes identified in our study were compared with those reported in the original article, and the clusters were named using the same markers: malignant cells (clusters 2, 3, 4, 8, and 19), cholangiocytes (cluster 13), endothelial cells (cluster 11), hepatocytes (cluster 14), fibroblasts (cluster16), B cells (clusters 17 and 18), T cells and natural killer (NK) cells (cluster 0, 1, 7, 9, and 10), and macrophages and dendritic cells (clusters 5, 6, 12, and 15). Cells derived from ICC or normal tissue are shown in Figure 2G. The heatmap displayed the top 85 differential genes in the 20 clusters (Figure 2H). Cluster maps were used to show the top four genes with significant correlation to each cluster (Supplementary Figures 2–5).

**Table 1:** In total, 33,991 cells from eight ICC patients were analyzed in this study.

**Patient**	**Cell count**	**Percentage (%)**
18N	10,317	30.35
18T	4,963	14.60
20T	2,714	7.98
23N	4,425	13.02
23T	3,504	10.31
24T1	3,468	10.20
24T2	2,441	7.18
25N	2,159	6.35
Total	33,991	100

**Figure 3:**
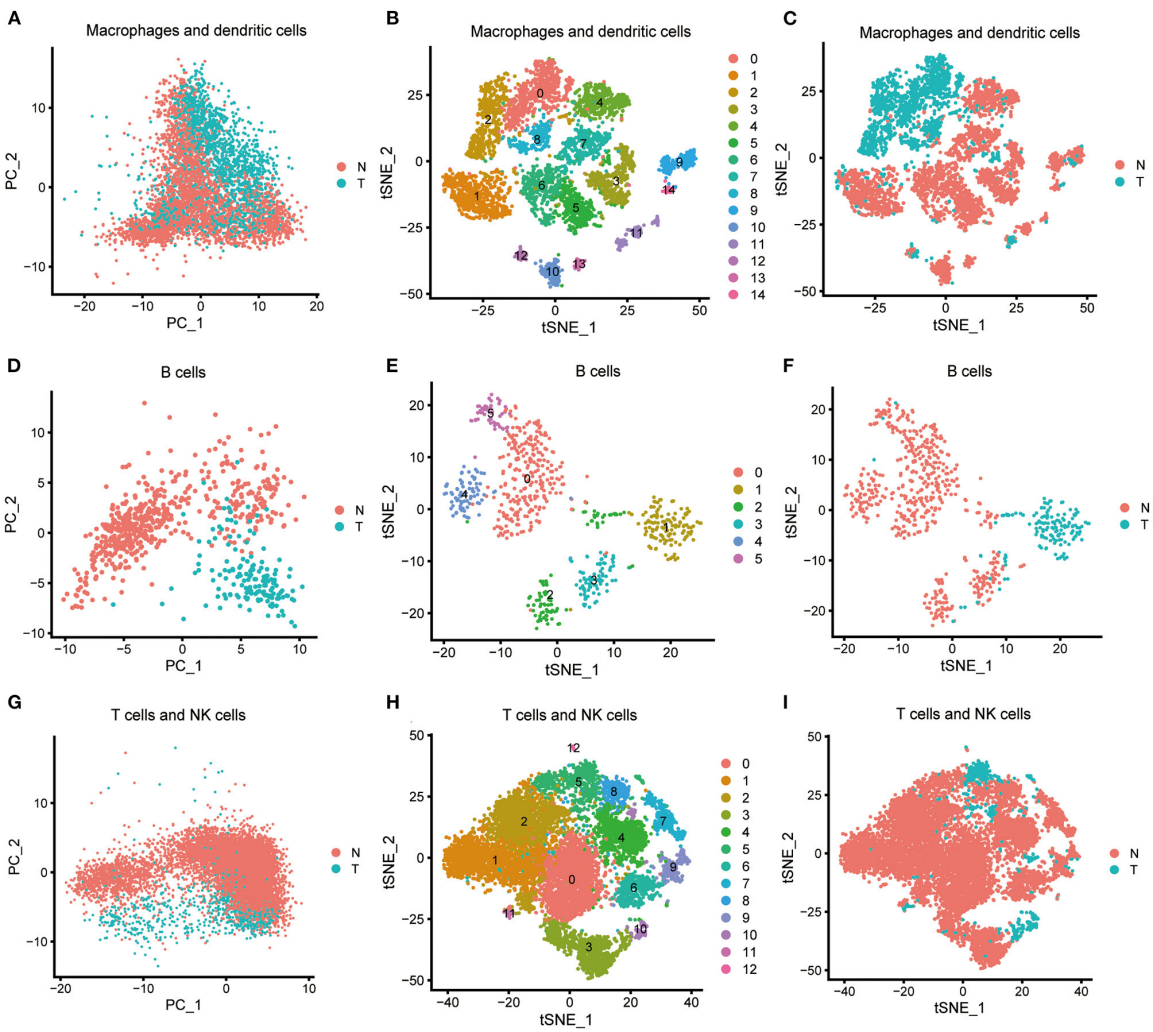
Characterization of scRNA-seq from immune cells and screening of marker genes. **(A)** Macrophages and dendritic cells were processed by PCA. **(B)** Macrophages and dendritic cells were further classified into 15 subclusters by t-SNE algorithm. Subclusters 5, 6, 10, 11, 12, and 13 were identified as dendritic cells; subclusters 0, 1, 2, 3, 4, 7, 8, 9, and 14 were identified as macrophages. **(C)** Macrophages and dendritic cells derived from ICC or normal tissue are shown. **(D)** B cells were processed by PCA. **(E)** B cells were further classified into six subclusters by the t-SNE algorithm. **(F)** B cells derived from ICC or normal tissue are shown. **(G)** T cells and NK cells were processed by PCA. **(H)** T cells and NK cells were further classified into 13 subclusters by the t-SNE algorithm. **(I)** T cells and NK cells derived from ICC or normal tissue are shown.

We processed the immune cells as described above. Macrophages and dendritic cells, consisting of clusters 5, 6, 12, and 15, were preliminarily processed using PCA (Figure 3A) and were further classified into 15 subclusters using the t-SNE algorithm (Figure 3B, Supplementary Table 2). Subclusters 5, 6, 10, 11, 12, and 13 were identified as dendritic cells, and the remaining subclusters were macrophages. The macrophages derived from ICC tissue could be easily distinguished from those derived from normal tissue (Figure 3C), and 337 macrophage marker genes were identified by comparing subclusters 0, 2, and 8 with subclusters 1, 3, 4, 7, 9, and 14. Other relevant single-cell analysis results are shown in Supplementary Figures 6, 7. We processed the clusters 17 and 18 B cells by PCA (Figure 3D) and successfully classified them into six subclusters using the t-SNE algorithm (Figure 3E, Supplementary Table 3). B cells derived from ICC tissue could be distinguished from those derived from normal tissue (Figure 3F). We compared subcluster 1 with subclusters 0, 2, 3, 4, and 5 and identified 427 B cell marker genes. Other relevant single-cell analysis results are displayed in Supplementary Figures 8, 9. T cells and NK cells, consisting of clusters 0, 1, 7, 9, and 10, were processed by PCA (Figure 3G) and further divided into 13 subclusters using the t-SNE algorithm (Figure 3H, Supplementary Table 4). The cells derived from ICC tissue could not be distinguished from those derived from normal tissue (Figure 3I). Therefore, we did not screen for marker genes in the different subclusters. Other relevant results are shown in Supplementary Figure 10. In total, 427 and 337 marker genes were identified in B cells and macrophages, respectively, and were merged together as DEGs. Finally, 659 DEGs were obtained (Supplementary Table 5).

### Development and Validation of a PRG Signature in ICC Populations

Gene expression data and clinical information from 33 ICC samples were acquired from the TCGA data portal, and transcriptome data and clinical data of 30 ICC samples were obtained from the GEO-ICC. The expression of 659 DEGs were extracted separately from TCGA-ICC and GEO-ICC and then merged with survival information. The consolidated TCGA-ICC and GEO-ICC clinical information is shown in Table 2. Fifteen DEGs with *P* < 0.05 were preliminary screened out in the training set using univariate COX analysis. Finally, nine PRGs were identified by multivariate Cox regression (Table 3).

**Table 2:** Clinical characteristics of TCGA-ICC and GEO-ICC samples in this study.

**Variables**	**Training group**	**Testing group**
	**(*N* = 33)**	**(*N* = 30)**
Age(Mean±SD)	—	65.60 ± 8.74
Follow-up(y)	1.99 ± 1.51	2.32 ± 1.74
**Status**		
Alive	18 (54.55)	13 (43.33)
Dead	15 (45.45)	17 (56.67)
**Gender**		
Male	14 (42.42)	24 (80.00)
Female	19 (57.58)	6 (20.00)
**Stage**		
I	18 (54.55)	15 (50.00)
II	9 (27.27)	6 (20.00)
III	1 (3.03)	1 (3.33)
IV	5 (15.15)	8 (26.67)
**AJCC-T**		
T1	18 (54.55)	—
T2	10 (30.30)	—
T3	5 (15.15)	—
T4	0	—
**Risk-score levels**		
High	16 (48.48)	18 (60.00)
low	17 (51.52)	12 (40.00)

**Table 3:** Univariate and multivariate analysis for OS in patients with ICC.

**Gene symbol**	**Univariate COX regression**		**Multivariate COX regression**		
	**HR (95% CI)**	***P***	**HR (95% CI)**	***P***	**Coefficient**
ANXA1	1.019 (1.003, 1.034)	0.019			
GLIPR1	1.076 (1.010, 1.146)	0.023			
TMEM107	1.295 (1.017, 1.648)	0.036			
MT2A	1.004 (1.001, 1.007)	0.009			
PMEPA1	1.009 (1.001, 1.018)	0.038			
VIM	1.002 (1.000, 1.003)	0.043			
SLC16A3	1.070 (1.016, 1.127)	0.010	0.686 (0.538, 0.875)	0.002	−0.377
BNIP3L	1.095 (1.004, 1.194)	0.041	1.239 (1.013, 1.516)	0.037	0.214
TPM2	1.014 (1.003, 1.024)	0.008	1.265 (1.109, 1.442)	0.000	0.235
CLEC11A	1.075 (1.001, 1.155)	0.046	0.767 (0.648, 0.909)	0.002	−0.265
EREG	1.135 (1.018, 1.266)	0.023	1.349 (0.956, 1.904)	0.088	0.300
PMAIP1	1.053 (1.004, 1.103)	0.033	0.397 (0.237, 0.665)	0.000	−0.925
CEBPB	1.020 (1.004, 1.036)	0.016	1.074 (1.032, 1.118)	0.000	0.072
A2M	1.008 (1.001, 1.015)	0.016	1.031 (1.015, 1.047)	0.000	0.030
TUBA1B	1.034 (1.007, 1.063)	0.014	1.129 (1.053, 1.211)	0.001	0.121

**Figure 4:**
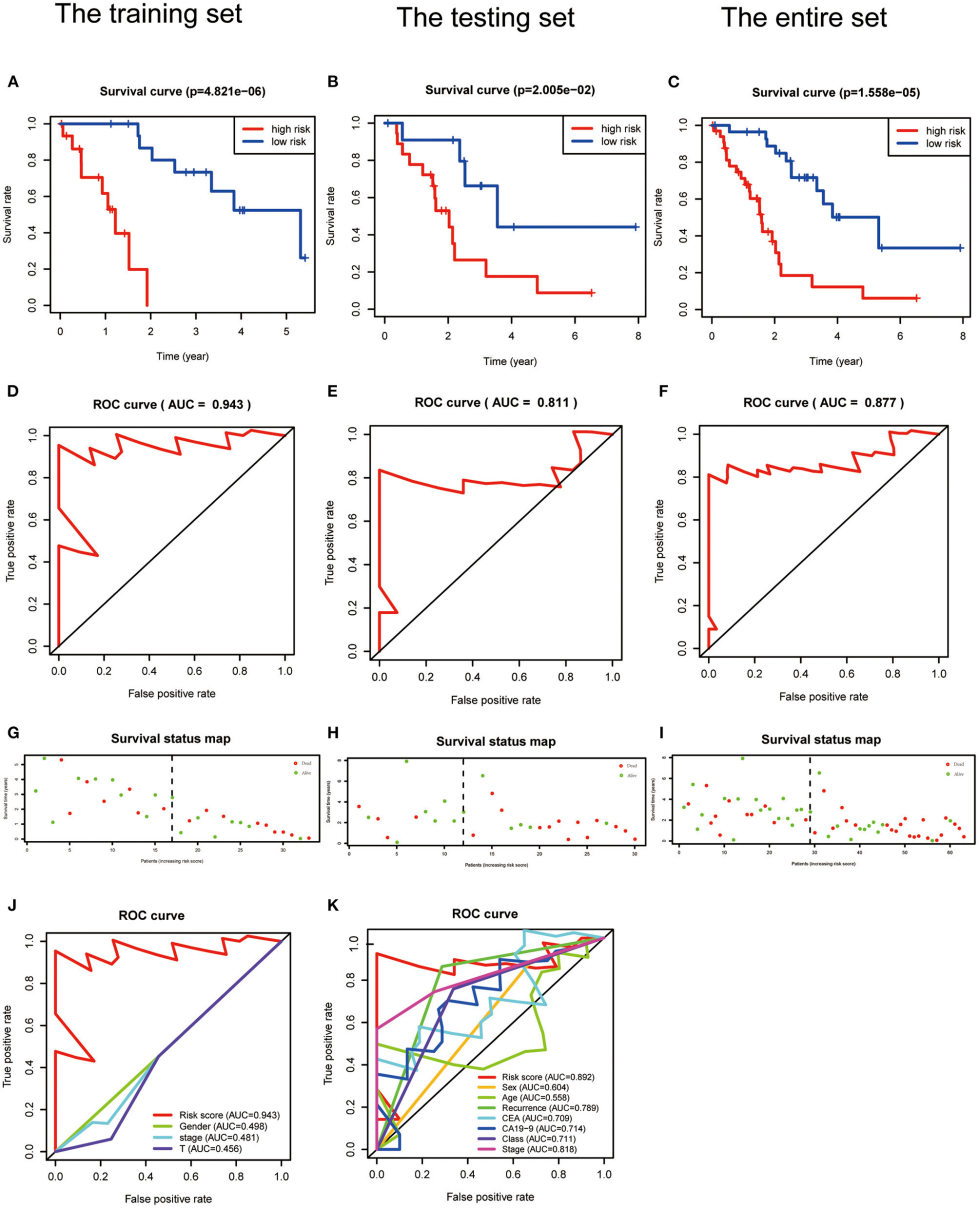
Validation of a PRGs signature for ICC. **(A–C)** Survival curves for the low- and high-risk groups of the training, testing, and entire sets. **(D–F)** ROC analysis predicted 3-year OS using the risk scores of the training, testing, and entire sets. **(G–I)** Survival status and duration of the training, testing, and the entire sets. **(J,K)** ROC curves validated the prognostic value of the PRGs and clinical characteristics at the 3-year level in the training and testing sets.

Kaplan–Meier analysis revealed that patients with high PRG risk scores suffered worse outcomes in the training set (Figure 4A, Supplementary Table 6). The predictive power of the signature was further validated in the testing set and the entire set (Figures 4B,C; Supplementary Table 7). Kaplan–Meier analysis with *P* < 0.05 for each PRG is shown in Supplementary Figures 11F–I. The AUCs for 3-year outcome prediction were 0.943, 0.811, and 0.877 in the training, testing, and entire sets (Figures 4D–F). These results suggest that the signature is sensitive. ROC curve analysis using the survival signature risk score of entire set for 1-, 2-, 4-, 5-, and 6-year survival is displayed in Supplementary Figures 11A–E. The survival status maps based on PRG risk scores were generated for each of the three sets, and the distribution plot shows that high risk scores were associated with increased death rates (Figures 4G–I).

### Comparison of the PRG Signature and Clinical Characteristics in the TCGA-ICC and GEO-ICC Cohorts

**Figure 5:**
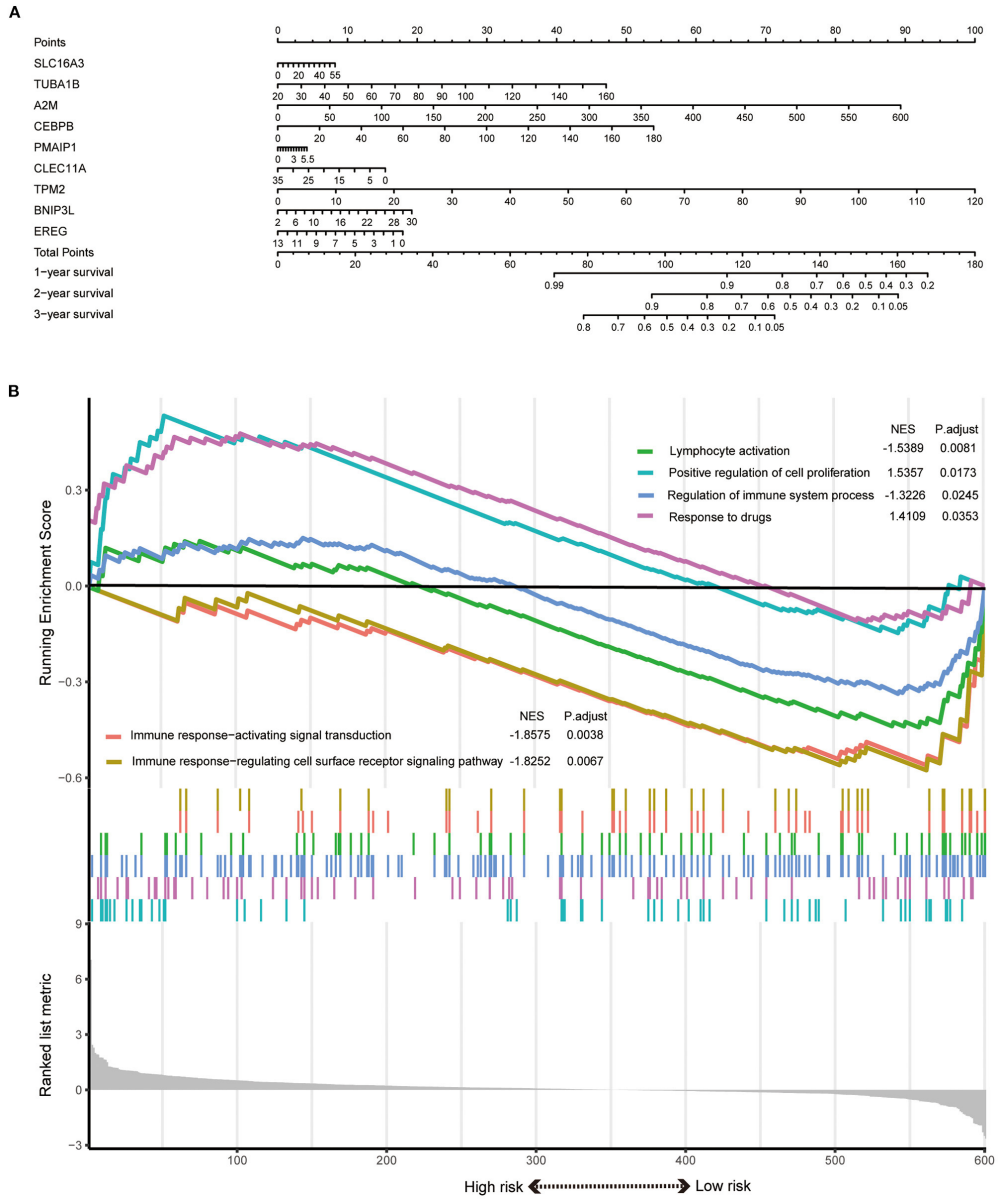
**(A)** A nomogram model was developed using the PRGs. **(B)** The significantly enriched biological processes between two risk score levels in the GSEA of GO.

The signature always get the greatest AUC value compared with the other clinical features at 3-year in the two sets, indicating its better predicting power (Figures 4J,K). The downloaded TCGA-ICC clinical data included only gender, tumor stage, and T stage information, so the correlation between risk score and the other clinical features was not analyzed. The downloaded GEO-ICC clinical data included sex, age, recurrence, CEA, CA-19-9, class, and stage. Nine PRGs were used to develop a nomogram model to predict OS for ICC (Figure 5A).

### Functional Enrichment Analysis Revealed Different States Between High- and Low-Risk Groups

GSEA results indicated that the response to drugs and positive regulation of cell proliferation were significantly associated with the high-risk phenotype. However, the regulation of immune system process, immune response-activating signal transduction, immune response-regulating cell surface receptor signaling pathway, and lymphocyte activation were enriched in the low-risk group (Figure 5B).

## Discussion

The liver cancer immune microenvironment includes almost all immune cell types (Dadi et al., 2016). The type, density, and location of immune cells have significant effects on patient prognosis and tumor evolution (Galon and Bruni, 2019). Some studies have illustrated the relationship between immune cells and ICC progression (Sulpice et al., 2016), but most of these studies screened for biomarkers by comparing tissue sequencing results of tumor and non-tumor tissues. Conventional tissue sequencing assays use a mixture of millions of cells, or more, and the result represents the average transcriptome expression of a group cells, or the information of the dominant cells. Using this approach means that there is the potential to miss significant genes. scRNA-seq is especially useful for tumors to precisely reflect the heterogeneity within the tumor cells (Ellsworth et al., 2017). The heterogeneous information between single cells can be uncovered (Nguyen et al., 2018), and the significant genes that truly characterize the cells in cancer can be discovered (Kulkarni et al., 2019). Therefore, PRG signatures, based on scRNA-seq data of immune cells, can be used as reliable biomarkers to predict prognosis in ICC.

In this study, the raw scRNA-seq data from 33,991 cells were analyzed. The cells and genes with poor sequencing quality were excluded and were a significant confounding factor for the statistical results. Only cells with high proportions were screened for further analysis, which ensured the reliability of the analysis results. The subsequent PCA was performed, which carried out a linear dimensionality reduction while maintaining data characteristics as much as possible. Next, the t-SNE algorithm was used to conduct non-linear dimensionality reduction. The combination of PCA and t-SNE algorithms ensured the efficiency and accuracy of dimension reduction. We only get a result that was very similar to the original paper, because the filter criteria and processing parameters were different from those in the original article. Clusters 0, 1, 7, 9, and 10 could roughly be identified as T cells and NK cells, because gene expression in these two cell types is almost the same. For our study, the marker genes provided in the original article were not specific enough to be used to further subdivide these clusters. We also noticed that T and NK cells derived from ICC tissue were mixed with those derived from normal tissue. Similarly, clusters 5, 6, 12, and 15 were roughly defined as macrophages and dendritic cells, but there was a relative boundary between normal and ICC clusters. The other clusters precisely identified the cell types, including cholangiocytes, endothelial cells, fibroblasts, hepatocytes, and malignant cells. Notably, clusters 17 and 18 were identified as B cells, and normal B cells were distinguishable from those derived from ICC. Finally, 659 marker genes were screened out as DEGs between the ICC and normal groups. These genes represented the internal heterogeneity of immune cells and might be closely related to OS in ICC.

DEGs were preliminarily screened by univariate Cox regression, and multivariate Cox regression was used to further optimize and select variables. Finally, a prognostic signature comprising nine PRGs was established. This signature was validated in the testing and entire sets, suggesting it was efficacious in predicting prognosis for ICC patients under diverse clinical conditions. The signature also had higher predictive accuracy and efficacy than did clinical characteristics. Finally, the nomogram model was developed and shows promising application value in clinical practice because of its simplicity and convenience. Whether the prognostic signature has potential predictive ability for drug therapy remains to be determined and deserves further investigation.

The nine PRGs in this nomogram play essential roles in the development and progression of many malignant tumors. SLC16A3 is associated with pancreatic cancer and glioblastoma and can predict tumor metastasis and survival in lung adenocarcinoma (Zhang et al., 2019; Yu et al., 2020). BNIP3L is a new prognostic biomarker for melanoma patients (Kazimierczak et al., 2020) and is related to the development and metastasis of hepatocellular carcinoma (HCC) and colon cancer (Chen et al., 2020). TPM2 is related to gastric cancer (Lin et al., 2019). CLEC11A is linked to the development of a variety of cancers, including leukemia, multiple myeloma, and gastrointestinal tumors (Wang et al., 2020). EREG is closely associated with the progression of various tumors, including gynecological cancer, rectal cancer, lung adenocarcinoma, breast cancer, and lung squamous cell carcinoma (Lin et al., 2020; Tao et al., 2020; Zayed, 2020). PMAIP1 is related to the development of ovarian cancer and spinal cord glioma cells (Zheng et al., 2019; Gov, 2020). CEBPB is associated with the development of various tumors, including osteosarcoma, gastric cancer, HCC, glioblastoma, and human acute myeloid leukemia (Lu et al., 2019; Jinesh et al., 2020). A2M was identified as a key gene associated with various tumors, including non–small cell lung cancer, bladder cancer, osteosarcoma, and HCC (Ma et al., 2019; Huang et al., 2020). TUBA1B was related to breast cancer, HCC, and Wilms tumor (Lou et al., 2020; Tian et al., 2020). These nine PRGs are involved in the pathogenesis and development of many tumors, supporting their potential as a powerful biomarker to predict ICC prognosis.

To study the underlying molecular mechanism of the gene signature prognostic effects, GSEA was conducted. The results revealed that the pathways were mainly enriched in multiple tumor immune mechanisms, the regulation of these pathways can change the tumor cells immune microenvironment and affect the proliferation and migration of tumor cells. Our results also deepen our understanding of ICC and show that these nine PRGs possess great predictive value.

This study had several advantages. First, this was the first study to explore the correlation between DEGs and ICC prognosis. Second, a novel prognostic signature comprising nine PRGs, which reflected the immune specificity of each ICC patient and accurately predicted prognosis, was constructed. However, there were still several limitations. First, the GEO and TCGA ICC cohorts were not large enough because ICC is rare (Esnaola et al., 2016). This may affect the statistical validity of our results and limit their robustness. Second, relevant basic experiments are still needed to verify the results and identify the specific mechanisms of action. In future experiments, we plan to coculture macrophages and ICC cells. We will regulate the expression of PRGs in macrophages and observe the proliferation, migration, and invasion of ICC cells. This will help to confirm our findings presented here.

In conclusion, this study established a PRG signature based on scRNA-seq data of immune cells for ICC patients. This signature reflects the TME immune status and provides new biomarkers for ICC prognosis.

## Data Availability Statement

The datasets analyzed for this study were acquired from GEO data portal (https://www.ncbi.nlm.nih.gov/geo/) and TCGA data portal (https://portal.gdc.cancer.gov/).

## Ethics Statement

Ethical review and approval was not required for the study on human participants in accordance with the local legislation and institutional requirements. Written informed consent for participation was not required for this study in accordance with the national legislation and the institutional requirements.

## Author Contributions

All authors have made a substantial, direct and intellectual contribution to the work, and approved it for publication.

## Conflict of Interest

The authors declare that the research was conducted in the absence of any commercial or financial relationships that could be construed as a potential conflict of interest.

## Supplementary Material

Supplementary Figure 1 The correlated genes in each component from PCA procedure were exhibited. **(A)** Correlation analysis of the top 20 relevant genes. **(B)** The top 30 significantly correlated genes displayed by cluster analysis across each component. Colors ranging from purple to golden yellow represent correlated gene expression levels from low to high. **(C)** The scree plot shows the amount of variation each PC captured from the data.

Supplementary Figure 2 Cluster map displaying the top four significant marker genes of each cluster. **(A)** cluster 0, **(B)** cluster 1, **(C)** cluster 2, **(D)** cluster 3, and **(E)** cluster 4.

Supplementary Figure 3 Cluster map displaying the top four significant marker genes of each cluster. **(A)** cluster 5, **(B)** cluster 6, **(C)** cluster 7, **(D)** cluster 8, and **(E)** cluster 9.

Supplementary Figure 4 Cluster map displaying the top four significant marker genes of each cluster. **(A)** cluster 10, **(B)** cluster 11, **(C)** cluster 12, **(D)** cluster 13, and **(E)** cluster 14.

Supplementary Figure 5 Cluster map displaying the top four significant marker genes of each cluster. **(A)** cluster 15, **(B)** cluster 16, **(C)** cluster 17, **(D)** cluster 18, and **(E)** cluster 19.

Supplementary Figure 6 Characterization of scRNA-seq from macrophages and dendritic cells. **(A)** scRNA-seq data quality control of macrophages and dendritic cells for ICC cell and normal cell samples. **(B)** There was a positive association between detected gene counts and sequencing depth. **(C)** In total, 1,500 gene symbols with significant differences across macrophages and dendritic cells were identified, and the characteristic variance diagram was drawn. **(D)** Jack straw plot showing *P* value distributions for each PC. **(E)** The scree plot displayed the amount of variation each PC captured from the data. **(F)** The top 24 marker genes across the 15 clusters are exhibited. **(G)** Correlation analysis of the top 20 relevant genes. **(H)** The top 30 significantly correlated genes by cluster analysis across each component. Colors ranging from purple to golden yellow represent the expression levels of correlated genes from low to high.

Supplementary Figure 7 Cluster map displaying the top six significant marker genes of macrophages between ICC and normal tissue. **(A)** Macrophages derived from ICC tissue. **(B)** Macrophages derived from normal tissue.

Supplementary Figure 8 Characterization of scRNA-seq from B cells. **(A)** Quality control of B cell scRNA-seq data. **(B)** There was a positive association between detected gene counts and sequencing depth. **(C)** In total, 1,500 gene symbols with significant differences across B cells were identified and the characteristic variance diagram was drawn. **(D)** Jack straw plot showing *P*-value distributions for each PC. **(E)** The scree plot displayed how much variation each PC captured from the data. **(F)** The top 27 marker genes across the six clusters are exhibited. **(G)** Correlation analysis of the top 20 relevant genes. **(H)** The top 30 significantly correlated genes by cluster analysis across each component. Colors ranging from purple to golden yellow represent the expression levels of correlated genes from low to high.

Supplementary Figure 9 Cluster map displaying the top six significant B cell marker genes between ICC and normal tissue. **(A)** B cells derived from ICC tissue. **(B)** B cells derived from normal tissue.

Supplementary Figure 10 Characterization of T cell and NK cell scRNA-seq. **(A)** scRNA-seq data quality control of T cells and NK cells for ICC cell and normal cell samples. **(B)** There was a positive association between detected gene counts and sequencing depth. **(C)** In total, 1,500 gene symbols with significant differences across T cells and NK cells were identified and the characteristic variance diagram was drawn. **(D)** Jack straw plot showing *P*-value distributions for each PC. **(E)** The scree plot displayed how much variation each PC captured from the data. **(F)** Correlation analysis of the top 20 relevant genes. **(G)** The top 30 significantly correlated genes by cluster analysis across each component. Colors ranging from purple to golden yellow represent the expression levels of correlated genes from low to high.

Supplementary Figure 11 **(A–E)** ROC analysis using the signature risk scores of the entire set at 1, 2, 4, 5, and 6 years. **(F,G)** Survival analysis with *P*-value < 0.05 using each of the PRGs in the training set. **(H,I)** Survival analysis with *P*-value < 0.05 using each of the PRGs in the testing set.

Supplementary Table 1 Cluster classification of 29263 cells from the t-SNE algorithm.

Supplementary Table 2 Cluster classification of macrophages and dendritic cells from the t-SNE algorithm.

Supplementary Table 3 Cluster classification of B cells from the t-SNE algorithm.

Supplementary Table 4 Cluster classification of T cells and NK cells from the t-SNE algorithm.

Supplementary Table 5 Differentially expressed genes in macrophages and B cells (n = 659).

Supplementary Table 6 Calculation of PRGs risk scores in training set.

Supplementary Table 7 Calculation of PRGs risk scores in testing set.

## Abbreviations

TME: tumor microenvironment
ICC: intrahepatic cholangiocarcinoma
scRNA-seq: single-cell RNA sequencing
DEGs: differentially expressed genes
PRGs: prognosis-related genes
OS: overall survival
PRG: prognosis-related gene
TCGA: The Cancer Genome Atlas
GEO: Gene Expression Omnibus
TCGA-ICC: intrahepatic cholangiocarcinoma samples from The Cancer Genome Atlas
GEO-ICC: intrahepatic cholangiocarcinoma samples from Gene Expression Omnibus
GSEA: gene set enrichment analysis
adjPval: adjustment of *P*-value
ROC: receiver operating characteristic
AUC: area under the curve
PC: principal component
PCA: principal component analysis
t-SNE: t-distributed stochastic neighbor embedding
HCC: hepatocellular carcinoma
AJCC: American Joint Committee on Cancer.
