# Supplementary figures and images for: Development of a Prognostic Signature Based on Single-Cell RNA Sequencing Data of Immune Cells in Intrahepatic Cholangiocarcinoma
Source: Front Genet. 2021 Feb 4;11:615680. doi: 10.3389/fgene.2020.615680 (PMC7890365; doi:10.3389/fgene.2020.615680)

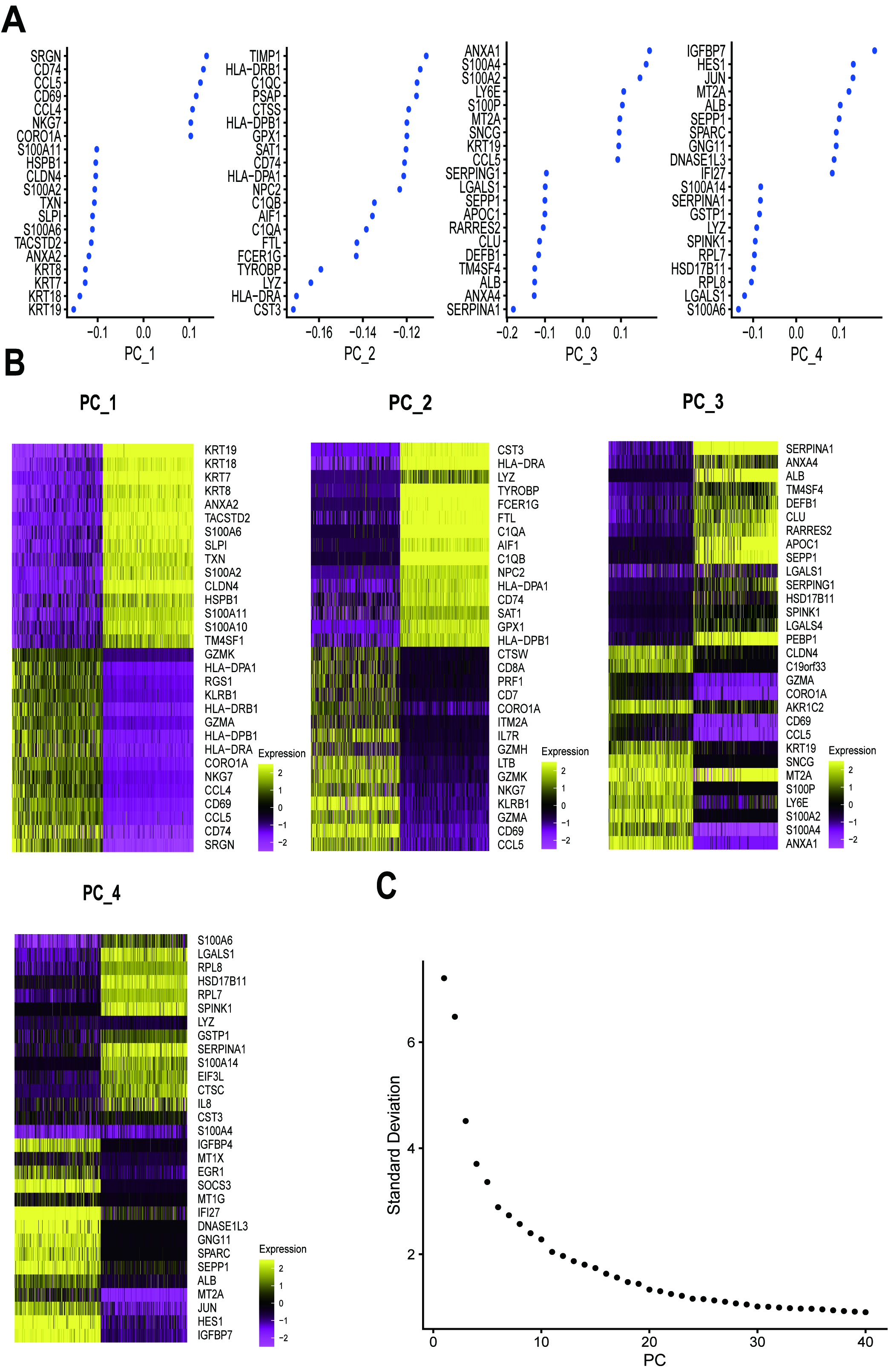

Supplement: Supplementary Figure 1 — The correlated genes in each component from PCA procedure were exhibited. (A) Correlation analysis of the top 20 relevant genes. (B) The top 30 significantly correlated genes displayed by cluster analysis across each component. Colors ranging from purple to golden yellow represent correlated gene expression levels from low to high. (C) The scree plot shows the amount of variation each PC captured from the data. [file Image_1.TIF]

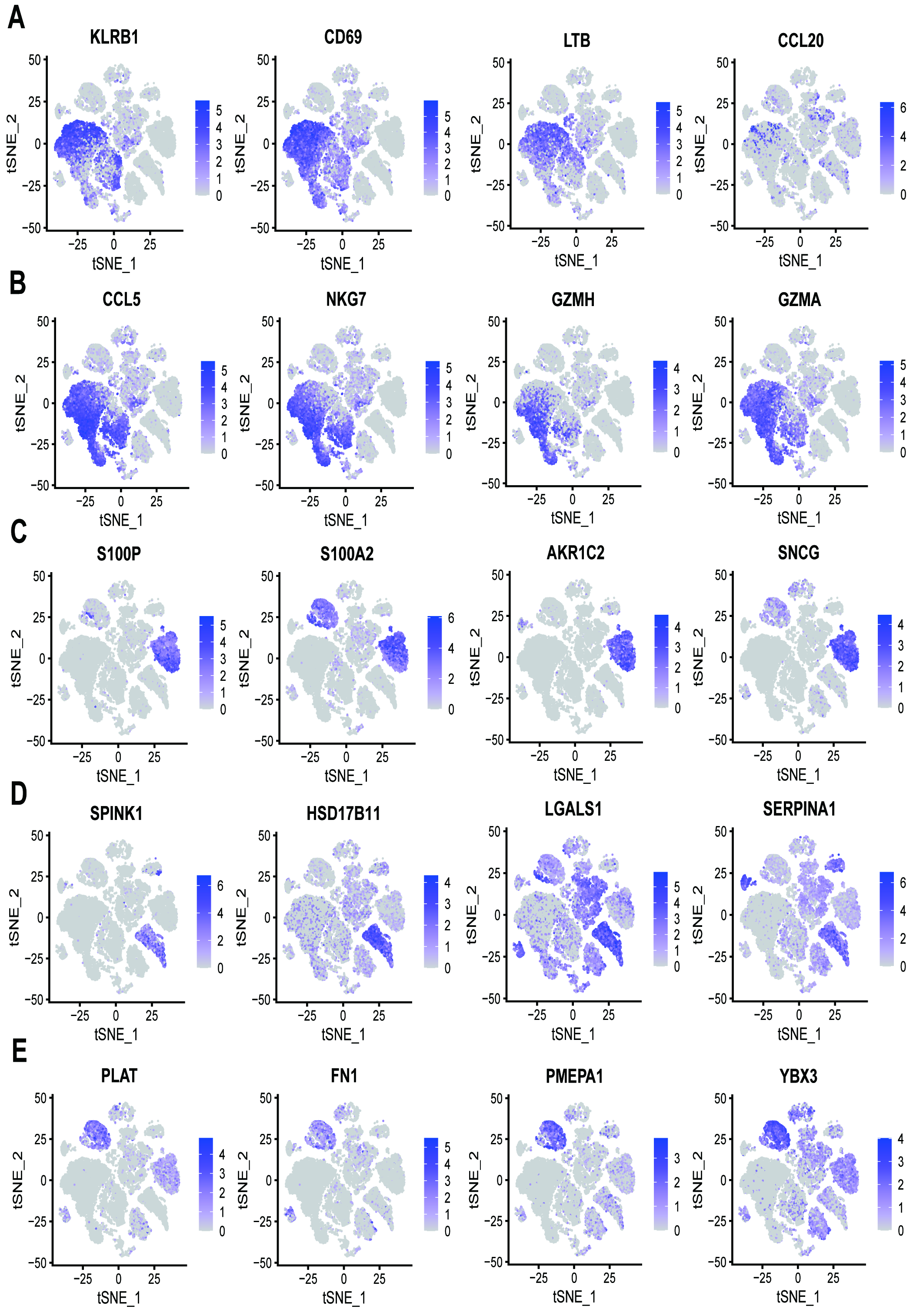

Supplement: Supplementary Figure 2 — Cluster map displaying the top four significant marker genes of each cluster. (A) cluster 0, (B) cluster 1, (C) cluster 2, (D) cluster 3, and (E) cluster 4. [file Image_2.TIF]

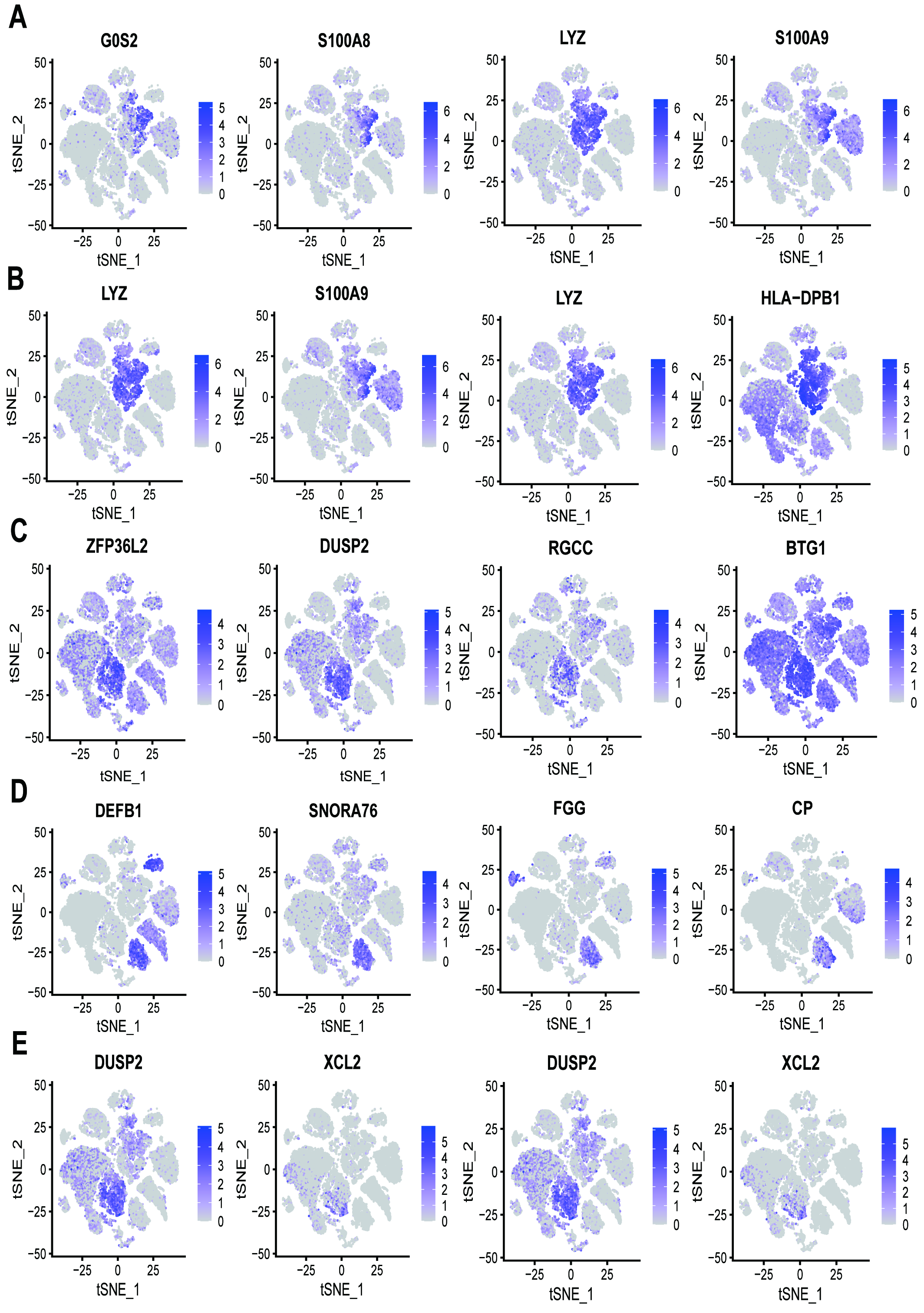

Supplement: Supplementary Figure 3 — Cluster map displaying the top four significant marker genes of each cluster. (A) cluster 5, (B) cluster 6, (C) cluster 7, (D) cluster 8, and (E) cluster 9. [file Image_3.TIF]

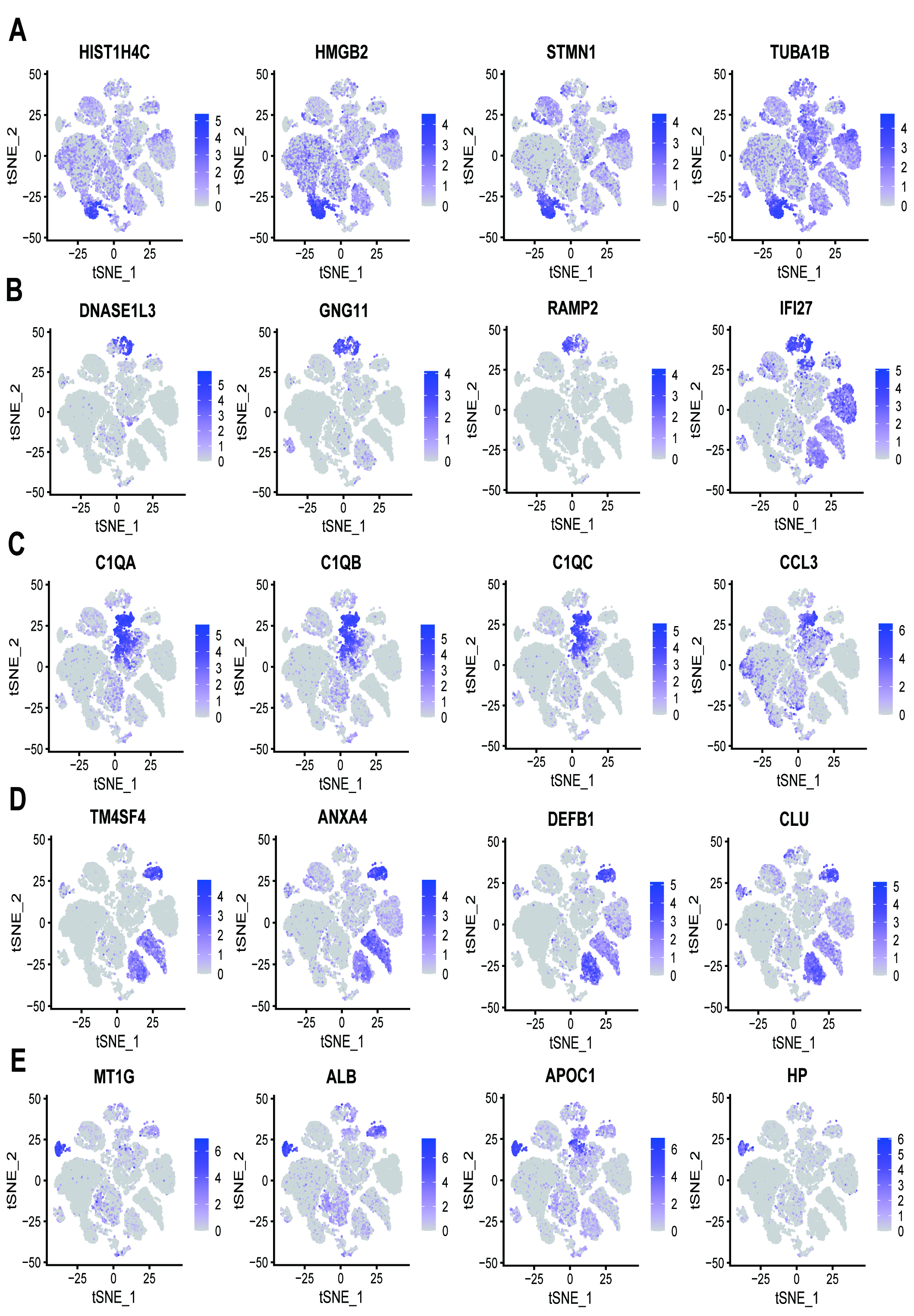

Supplement: Supplementary Figure 4 — Cluster map displaying the top four significant marker genes of each cluster. (A) cluster 10, (B) cluster 11, (C) cluster 12, (D) cluster 13, and (E) cluster 14. [file Image_4.TIF]

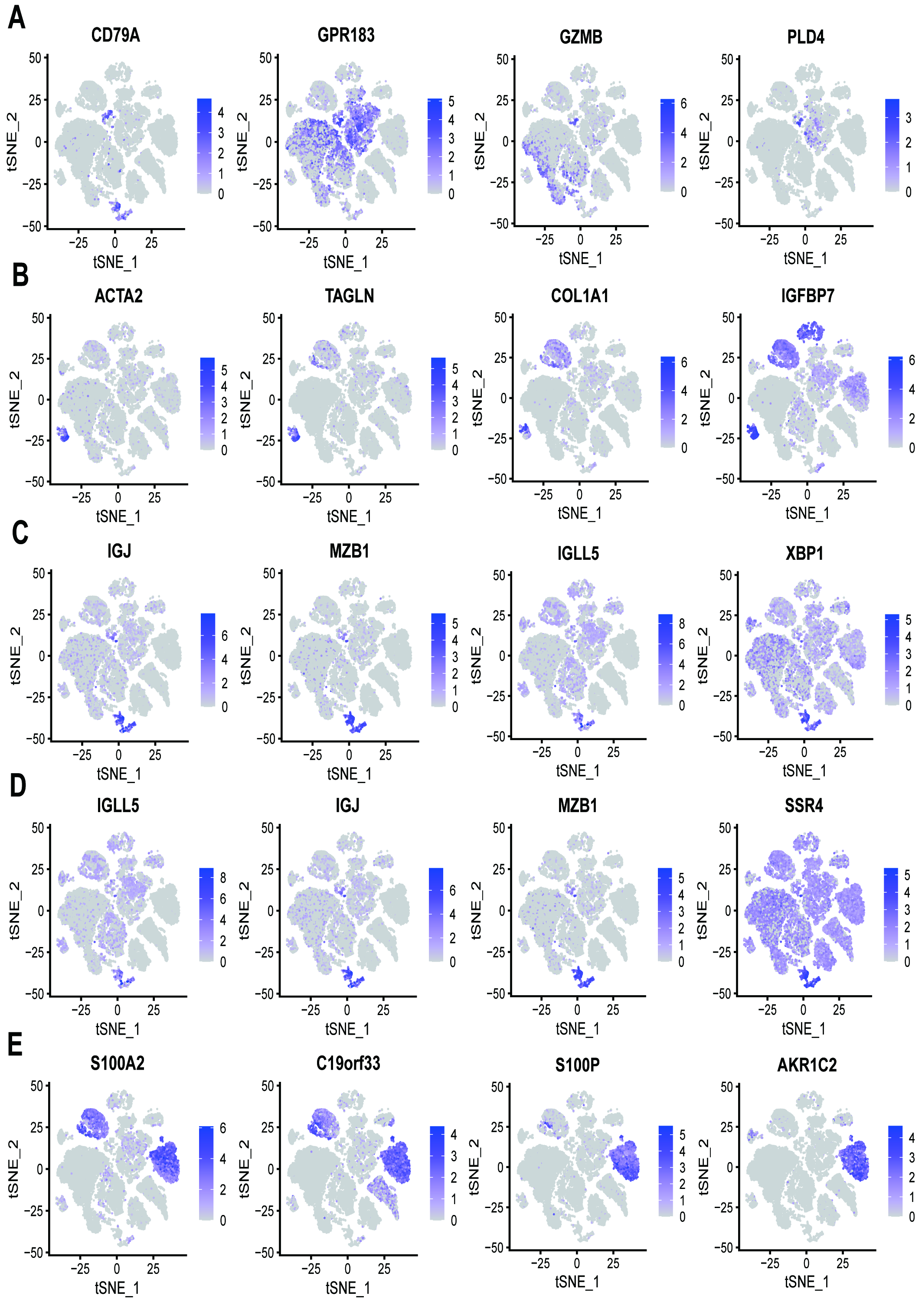

Supplement: Supplementary Figure 5 — Cluster map displaying the top four significant marker genes of each cluster. (A) cluster 15, (B) cluster 16, (C) cluster 17, (D) cluster 18, and (E) cluster 19. [file Image_5.TIF]

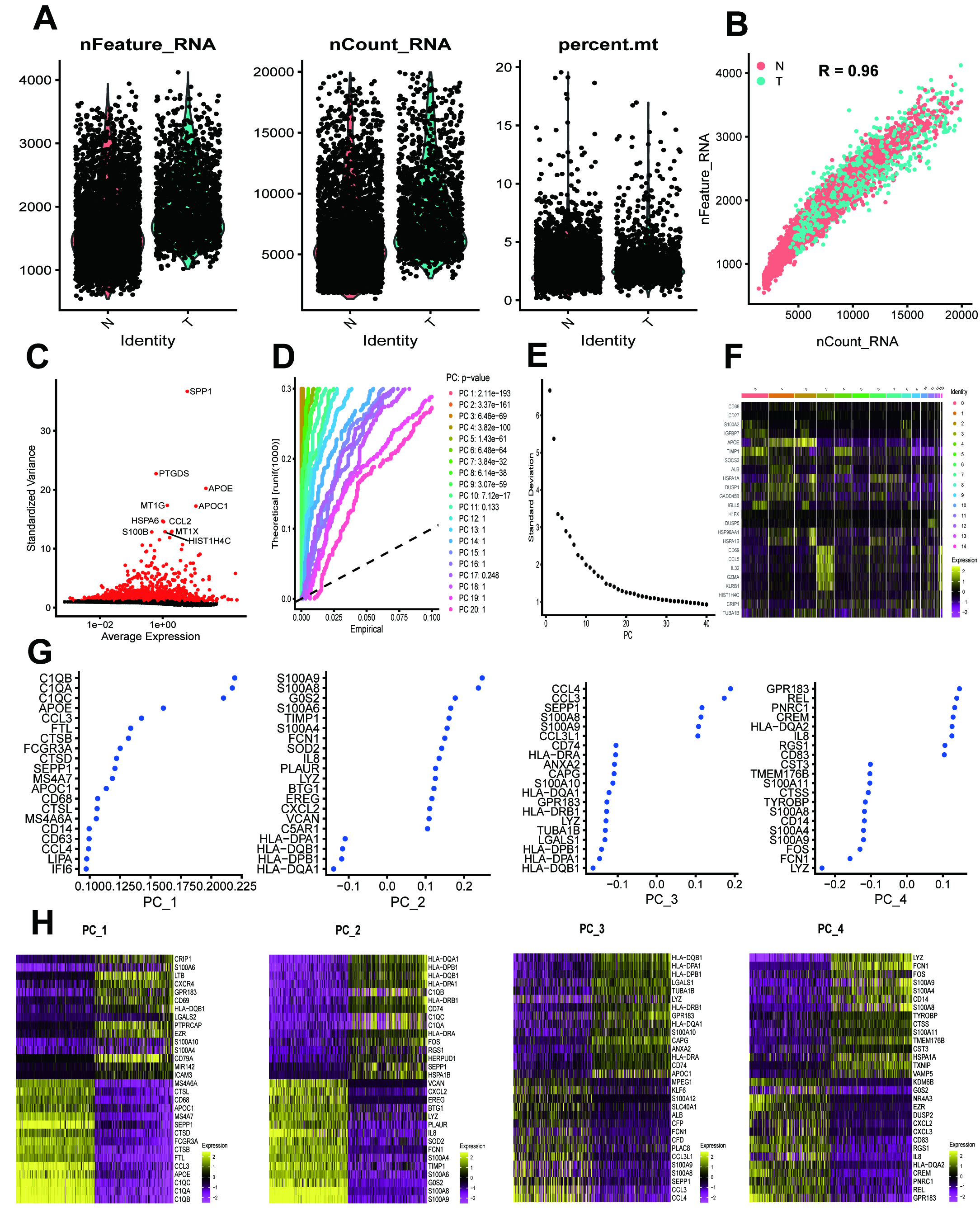

Supplement: Supplementary Figure 6 — Characterization of scRNA-seq from macrophages and dendritic cells. (A) scRNA-seq data quality control of macrophages and dendritic cells for ICC cell and normal cell samples. (B) There was a positive association between detected gene counts and sequencing depth. (C) In total, 1,500 gene symbols with significant differences across macrophages and dendritic cells were identified, and the characteristic variance diagram was drawn. (D) Jack straw plot showing P value distributions for each PC. (E) The scree plot displayed the amount of variation each PC captured from the data. (F) The top 24 marker genes across the 15 clusters are exhibited. (G) Correlation analysis of the top 20 relevant genes. (H) The top 30 significantly correlated genes by cluster analysis across each component. Colors ranging from purple to golden yellow represent the expression levels of correlated genes from low to high. [file Image_6.TIF]

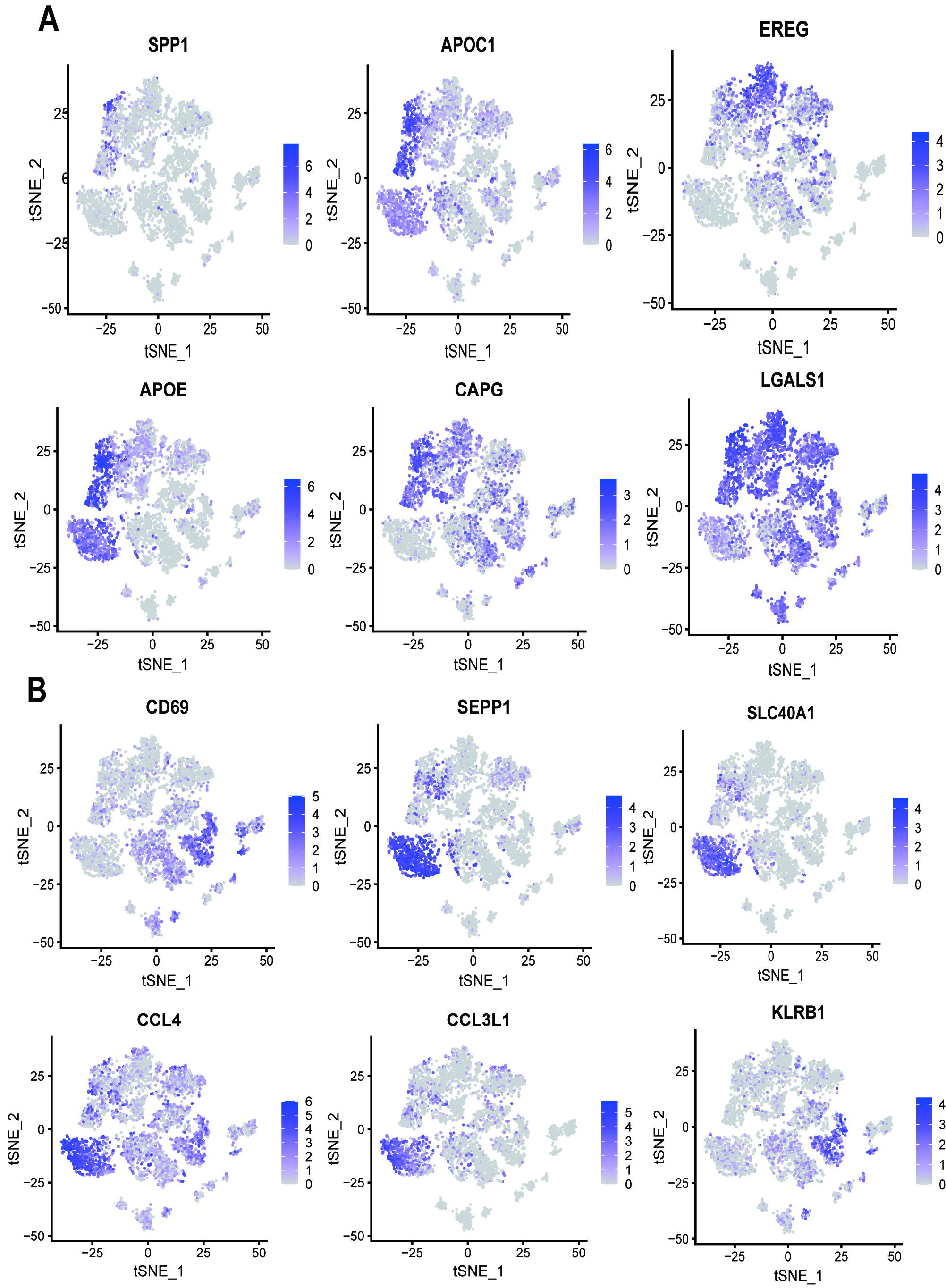

Supplement: Supplementary Figure 7 — Cluster map displaying the top six significant marker genes of macrophages between ICC and normal tissue. (A) Macrophages derived from ICC tissue. (B) Macrophages derived from normal tissue. [file Image_7.TIF]

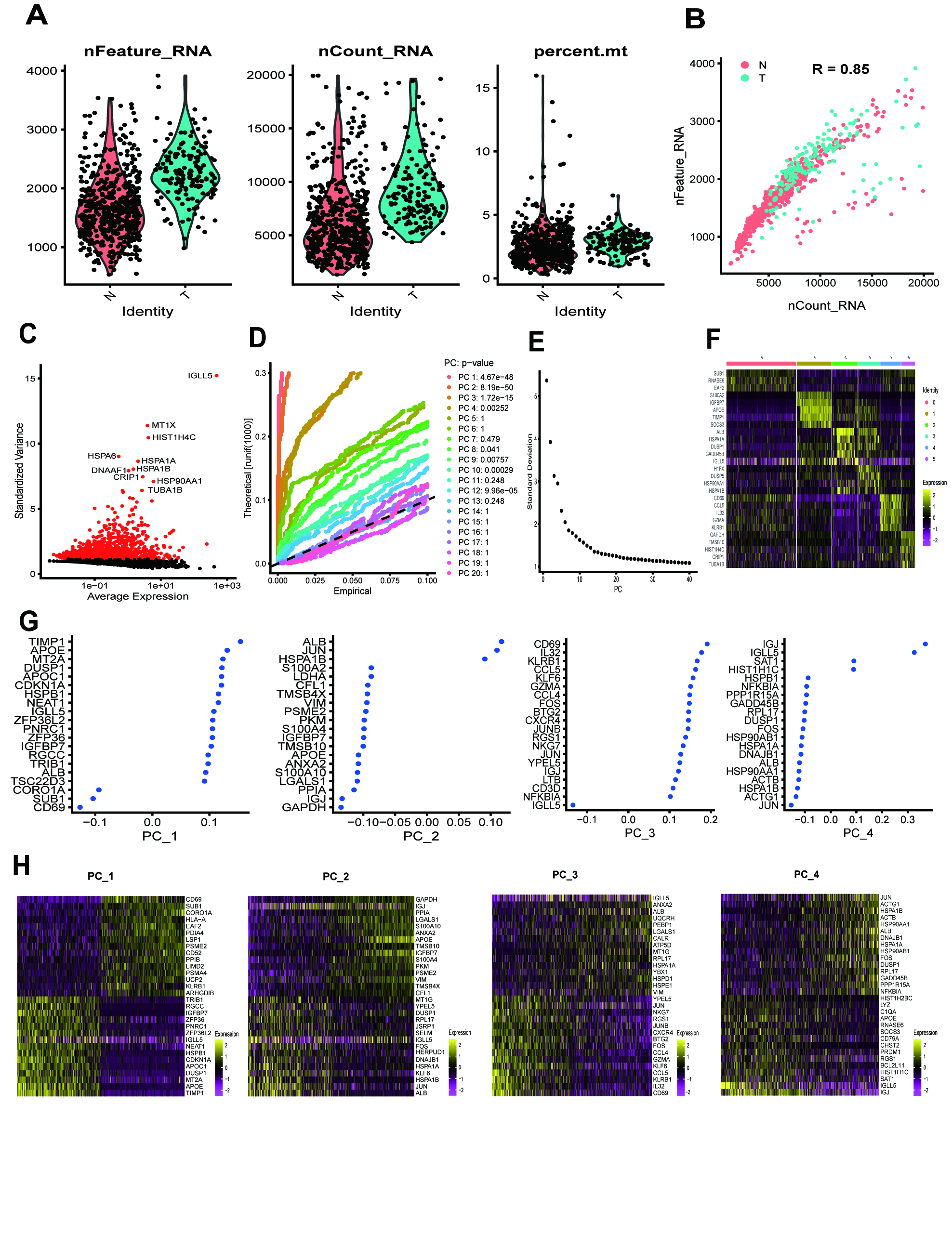

Supplement: Supplementary Figure 8 — Characterization of scRNA-seq from B cells. (A) Quality control of B cell scRNA-seq data. (B) There was a positive association between detected gene counts and sequencing depth. (C) In total, 1,500 gene symbols with significant differences across B cells were identified and the characteristic variance diagram was drawn. (D) Jack straw plot showing P-value distributions for each PC. (E) The scree plot displayed how much variation each PC captured from the data. (F) The top 27 marker genes across the six clusters are exhibited. (G) Correlation analysis of the top 20 relevant genes. (H) The top 30 significantly correlated genes by cluster analysis across each component. Colors ranging from purple to golden yellow represent the expression levels of correlated genes from low to high. [file Image_8.TIF]

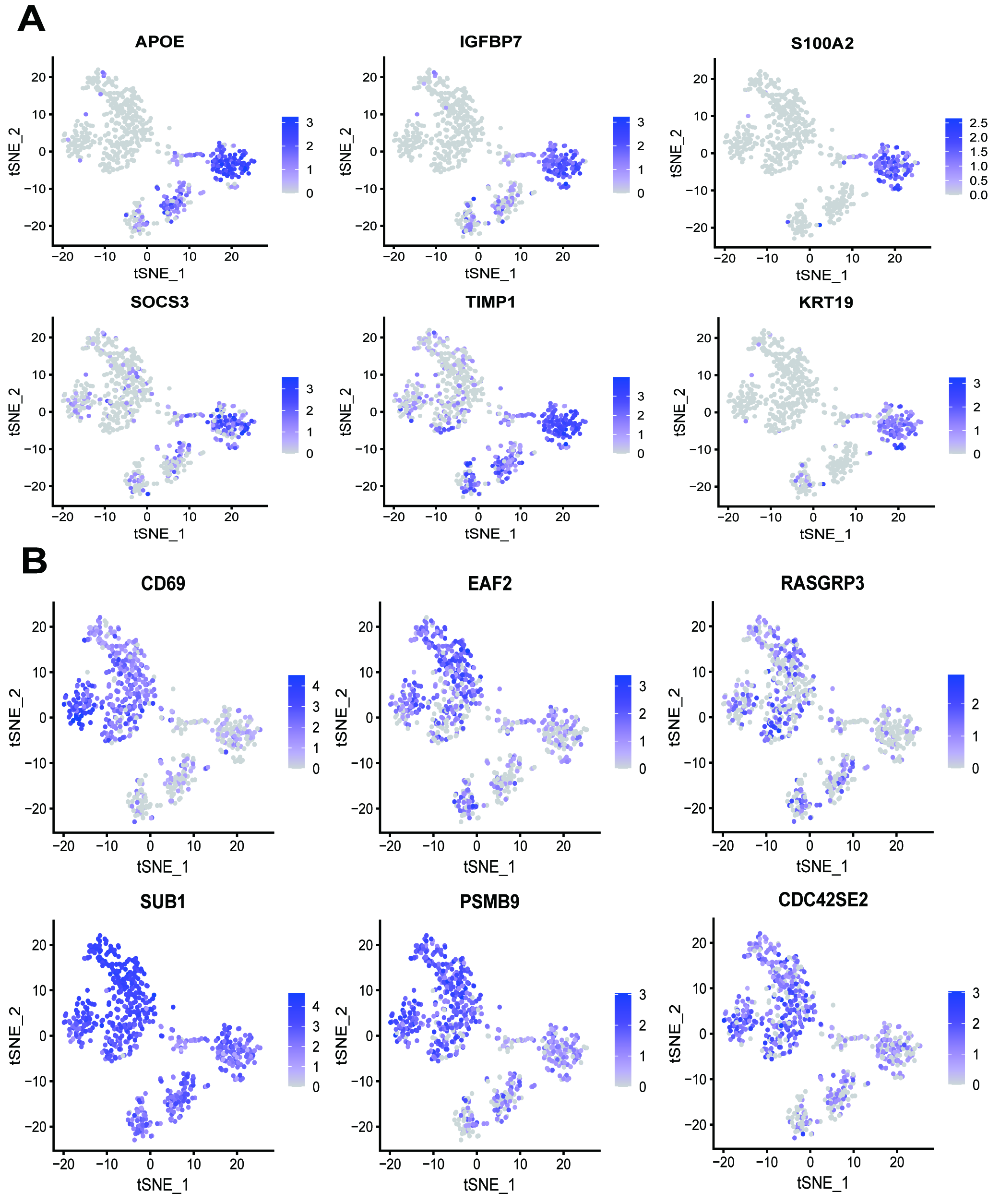

Supplement: Supplementary Figure 9 — Cluster map displaying the top six significant B cell marker genes between ICC and normal tissue. (A) B cells derived from ICC tissue. (B) B cells derived from normal tissue. [file Image_9.TIF]

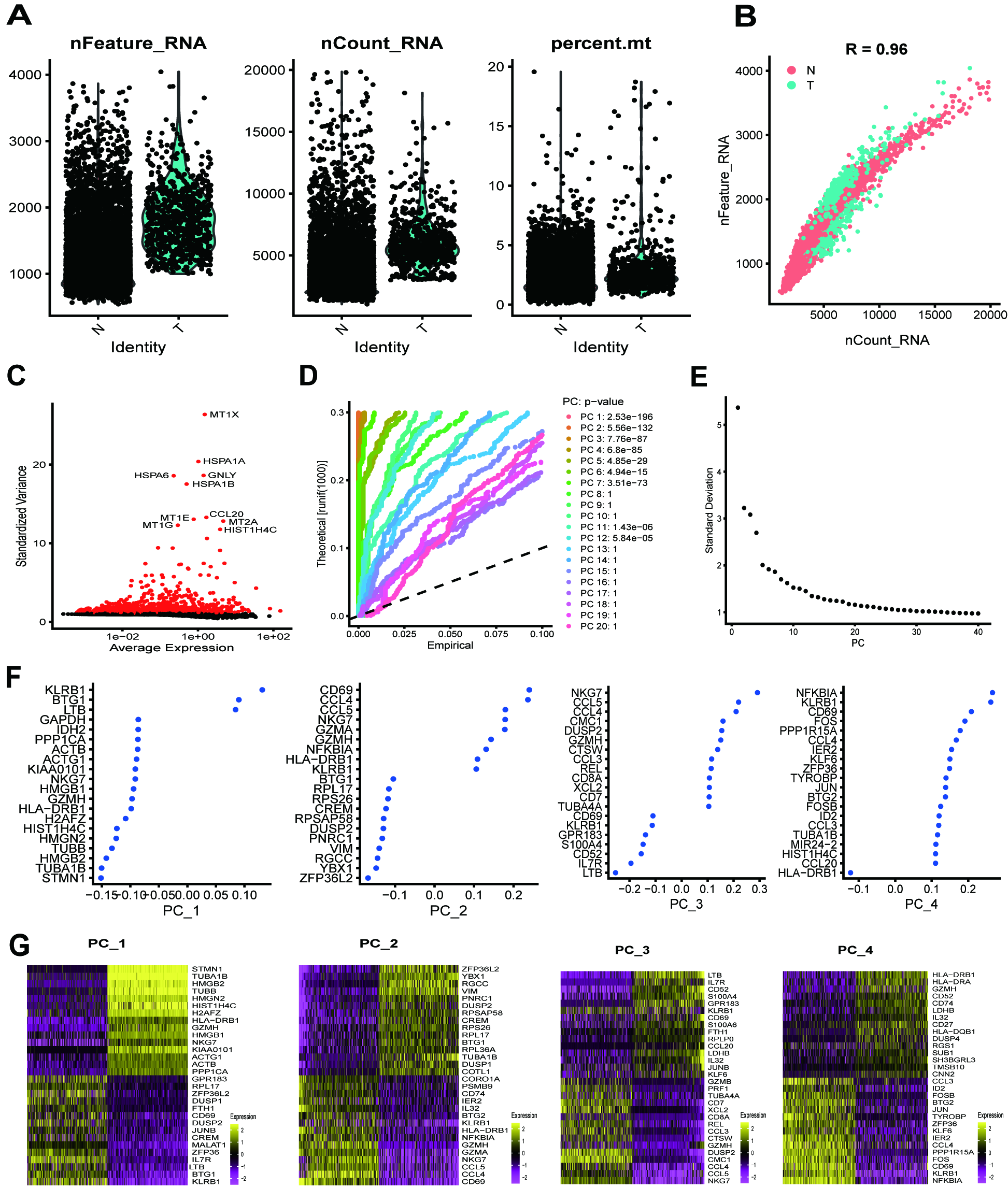

Supplement: Supplementary Figure 10 — Characterization of T cell and NK cell scRNA-seq. (A) scRNA-seq data quality control of T cells and NK cells for ICC cell and normal cell samples. (B) There was a positive association between detected gene counts and sequencing depth. (C) In total, 1,500 gene symbols with significant differences across T cells and NK cells were identified and the characteristic variance diagram was drawn. (D) Jack straw plot showing P-value distributions for each PC. (E) The scree plot displayed how much variation each PC captured from the data. (F) Correlation analysis of the top 20 relevant genes. (G) The top 30 significantly correlated genes by cluster analysis across each component. Colors ranging from purple to golden yellow represent the expression levels of correlated genes from low to high. [file Image_10.TIF]

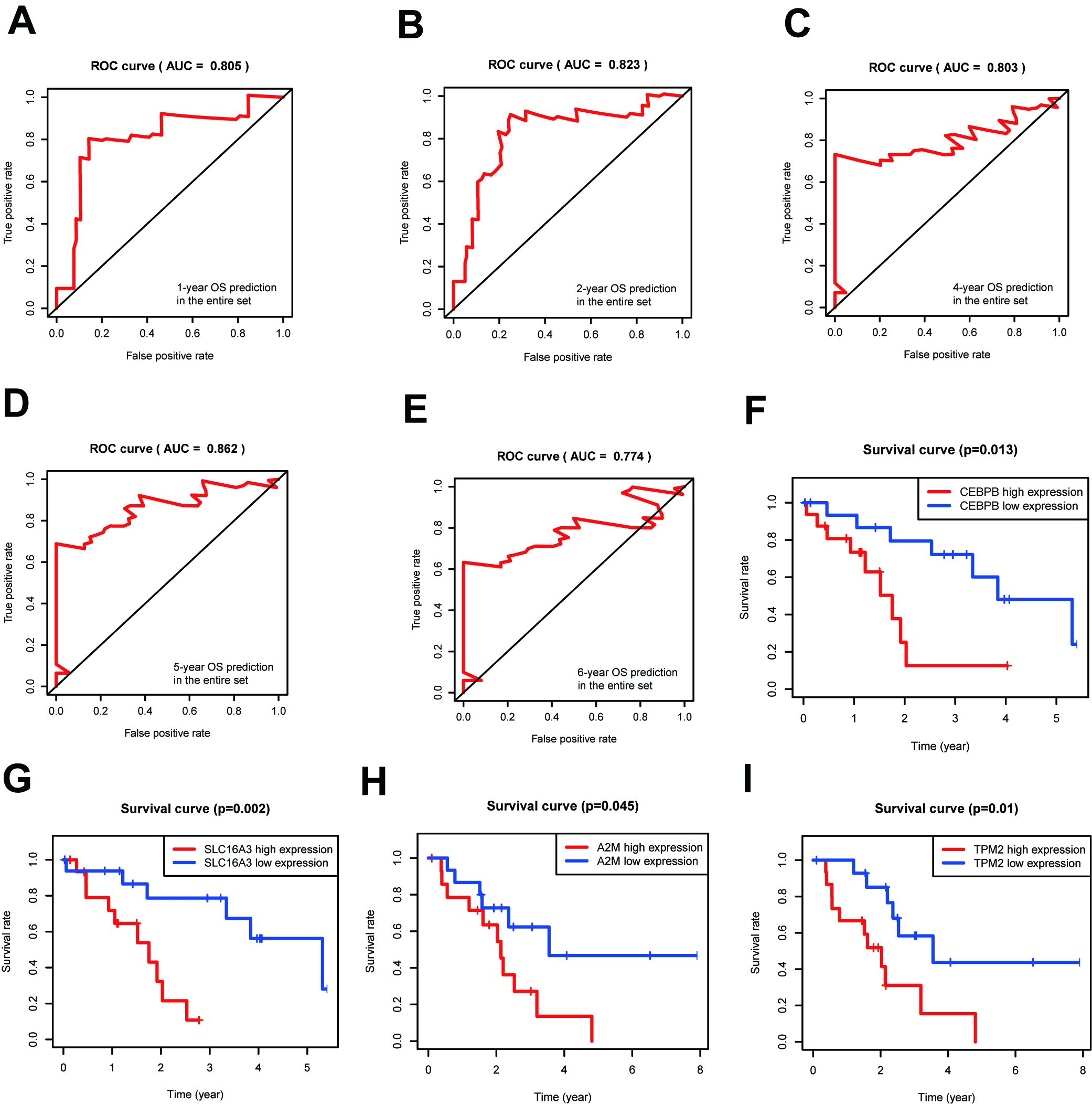

Supplement: Supplementary Figure 11 — (A–E) ROC analysis using the signature risk scores of the entire set at 1, 2, 4, 5, and 6 years. (F,G) Survival analysis with P-value < 0.05 using each of the PRGs in the training set. (H,I) Survival analysis with P-value < 0.05 using each of the PRGs in the testing set. [file Image_11.TIF]
